# Supplementary material for: Modeling glioblastoma heterogeneity as a dynamic network of cell states
Source: Mol Syst Biol. 2021 Sep 16;17(9):e10105. doi: 10.15252/msb.202010105 (PMC8444284; doi:10.15252/msb.202010105)
Supplement: Supplementary file 6 — Source Data for Figure 5 [file MSB-17-e10105-s004.zip › Figure5A_sourcedata/GSEA_3071/nosigdb_stateIV.GseaPreranked.1622795164626/FELDMAN_2020_S.html]

Details for gene set FELDMAN\_2020\_S[GSEA]

|  || Dataset | state53071 |
| Phenotype | NoPhenotypeAvailable |
| Upregulated in class | na\_neg |
| GeneSet | FELDMAN\_2020\_S |
| Enrichment Score (ES) | -0.55545217 |
| Normalized Enrichment Score (NES) | -3.035439 |
| Nominal p-value | 0.0 |
| FDR q-value | 0.0 |
| FWER p-Value | 0.0 |
Table: GSEA Results Summary

  

Fig 1: Enrichment plot: FELDMAN\_2020\_S      
 Profile of the Running ES Score & Positions of GeneSet Members on the Rank Ordered List

  

| PROBE | GENE SYMBOL | GENE\_TITLE | RANK IN GENE LIST | RANK METRIC SCORE | RUNNING ES | CORE ENRICHMENT || 1 | SCRG1 |  |  | 28 | 2.056 | 0.0134 | No |
| 2 | CDCA7L |  |  | 185 | 1.292 | -0.0051 | No |
| 3 | NOVA1 |  |  | 234 | 1.181 | -0.0036 | No |
| 4 | LYPD1 |  |  | 338 | 0.980 | -0.0146 | No |
| 5 | BEX4 |  |  | 341 | 0.980 | -0.0060 | No |
| 6 | ZFAND6 |  |  | 344 | 0.978 | 0.0026 | No |
| 7 | SOX4 |  |  | 377 | 0.928 | 0.0048 | No |
| 8 | SESN3 |  |  | 380 | 0.925 | 0.0129 | No |
| 9 | TGIF1 |  |  | 402 | 0.906 | 0.0171 | No |
| 10 | ARL6IP1 |  |  | 458 | 0.849 | 0.0142 | No |
| 11 | TSC22D1 |  |  | 588 | 0.735 | -0.0041 | No |
| 12 | PAIP2 |  |  | 658 | 0.683 | -0.0112 | No |
| 13 | MALAT1 |  |  | 739 | 0.638 | -0.0209 | No |
| 14 | CDCA7 |  |  | 818 | 0.596 | -0.0305 | No |
| 15 | SAT1 |  |  | 859 | 0.575 | -0.0330 | No |
| 16 | BTG1 |  |  | 1123 | 0.486 | -0.0795 | No |
| 17 | MARCKS |  |  | 1713 | 0.348 | -0.1905 | No |
| 18 | CARHSP1 |  |  | 2185 | 0.270 | -0.2793 | No |
| 19 | PTTG1 |  |  | 2470 | -0.266 | -0.3319 | No |
| 20 | LMNB1 |  |  | 2487 | -0.268 | -0.3325 | No |
| 21 | EIF1AX |  |  | 2584 | -0.280 | -0.3486 | No |
| 22 | RNASEH2A |  |  | 2595 | -0.282 | -0.3479 | No |
| 23 | ARL6IP6 |  |  | 2877 | -0.316 | -0.3995 | No |
| 24 | RMI2 |  |  | 2893 | -0.318 | -0.3995 | No |
| 25 | HNRNPAB |  |  | 2944 | -0.324 | -0.4062 | No |
| 26 | MSH6 |  |  | 3051 | -0.339 | -0.4237 | No |
| 27 | RAD51 |  |  | 3233 | -0.365 | -0.4554 | No |
| 28 | DCTPP1 |  |  | 3414 | -0.393 | -0.4867 | No |
| 29 | HAUS8 |  |  | 3443 | -0.398 | -0.4885 | No |
| 30 | TFDP1 |  |  | 3526 | -0.415 | -0.5006 | No |
| 31 | ACAT2 |  |  | 3695 | -0.443 | -0.5291 | No |
| 32 | HSPA8 |  |  | 3720 | -0.449 | -0.5296 | No |
| 33 | PGP |  |  | 3725 | -0.450 | -0.5263 | No |
| 34 | CALD1 |  |  | 3763 | -0.458 | -0.5293 | No |
| 35 | BAMBI |  |  | 3767 | -0.458 | -0.5257 | No |
| 36 | HES4 |  |  | 3779 | -0.461 | -0.5236 | No |
| 37 | DERA |  |  | 3799 | -0.466 | -0.5230 | No |
| 38 | RAB13 |  |  | 3933 | -0.494 | -0.5443 | No |
| 39 | SMC3 |  |  | 3952 | -0.498 | -0.5432 | No |
| 40 | MAGOHB |  |  | 3992 | -0.509 | -0.5461 | No |
| 41 | SLBP |  |  | 3994 | -0.509 | -0.5417 | No |
| 42 | YWHAH |  |  | 4032 | -0.521 | -0.5441 | No |
| 43 | TEX30 |  |  | 4076 | -0.532 | -0.5475 | No |
| 44 | CCNB2 |  |  | 4103 | -0.542 | -0.5476 | No |
| 45 | CACYBP |  |  | 4109 | -0.542 | -0.5436 | No |
| 46 | NCL |  |  | 4171 | -0.560 | -0.5503 | Yes |
| 47 | PPIF |  |  | 4178 | -0.561 | -0.5464 | Yes |
| 48 | EXOSC9 |  |  | 4189 | -0.564 | -0.5432 | Yes |
| 49 | MIS18A |  |  | 4230 | -0.575 | -0.5456 | Yes |
| 50 | ANP32B |  |  | 4235 | -0.576 | -0.5412 | Yes |
| 51 | HMGB2 |  |  | 4243 | -0.578 | -0.5372 | Yes |
| 52 | SNRPD1 |  |  | 4254 | -0.582 | -0.5339 | Yes |
| 53 | EEF1E1 |  |  | 4257 | -0.583 | -0.5289 | Yes |
| 54 | SIVA1 |  |  | 4263 | -0.586 | -0.5245 | Yes |
| 55 | MCM6 |  |  | 4273 | -0.590 | -0.5209 | Yes |
| 56 | EXOSC8 |  |  | 4293 | -0.596 | -0.5191 | Yes |
| 57 | ASF1B |  |  | 4328 | -0.606 | -0.5202 | Yes |
| 58 | RFC4 |  |  | 4340 | -0.611 | -0.5167 | Yes |
| 59 | FTL |  |  | 4350 | -0.615 | -0.5129 | Yes |
| 60 | DNMT1 |  |  | 4388 | -0.632 | -0.5143 | Yes |
| 61 | C19orf48 |  |  | 4427 | -0.644 | -0.5157 | Yes |
| 62 | EIF4EBP1 |  |  | 4474 | -0.667 | -0.5185 | Yes |
| 63 | RPA3 |  |  | 4482 | -0.670 | -0.5138 | Yes |
| 64 | PA2G4 |  |  | 4497 | -0.678 | -0.5103 | Yes |
| 65 | DHFR |  |  | 4517 | -0.688 | -0.5077 | Yes |
| 66 | EIF2S1 |  |  | 4553 | -0.707 | -0.5080 | Yes |
| 67 | CCNE2 |  |  | 4564 | -0.714 | -0.5034 | Yes |
| 68 | RFC2 |  |  | 4588 | -0.729 | -0.5012 | Yes |
| 69 | PTMA |  |  | 4620 | -0.746 | -0.5004 | Yes |
| 70 | RPA2 |  |  | 4638 | -0.760 | -0.4968 | Yes |
| 71 | DUT |  |  | 4642 | -0.762 | -0.4904 | Yes |
| 72 | USP1 |  |  | 4649 | -0.763 | -0.4846 | Yes |
| 73 | GGCT |  |  | 4666 | -0.772 | -0.4806 | Yes |
| 74 | MSH2 |  |  | 4673 | -0.781 | -0.4746 | Yes |
| 75 | CENPA |  |  | 4683 | -0.788 | -0.4692 | Yes |
| 76 | CDC45 |  |  | 4702 | -0.798 | -0.4654 | Yes |
| 77 | MCM7 |  |  | 4704 | -0.800 | -0.4583 | Yes |
| 78 | RANBP1 |  |  | 4741 | -0.823 | -0.4577 | Yes |
| 79 | RAN |  |  | 4743 | -0.824 | -0.4504 | Yes |
| 80 | CHAF1A |  |  | 4751 | -0.828 | -0.4442 | Yes |
| 81 | POLD3 |  |  | 4752 | -0.830 | -0.4366 | Yes |
| 82 | VRK1 |  |  | 4773 | -0.842 | -0.4328 | Yes |
| 83 | MCM5 |  |  | 4792 | -0.853 | -0.4285 | Yes |
| 84 | HELLS |  |  | 4819 | -0.882 | -0.4254 | Yes |
| 85 | HSPB11 |  |  | 4820 | -0.882 | -0.4174 | Yes |
| 86 | MCM3 |  |  | 4834 | -0.901 | -0.4117 | Yes |
| 87 | SMC2 |  |  | 4847 | -0.912 | -0.4057 | Yes |
| 88 | DLGAP5 |  |  | 4857 | -0.922 | -0.3990 | Yes |
| 89 | ORC6 |  |  | 4865 | -0.931 | -0.3918 | Yes |
| 90 | GMNN |  |  | 4873 | -0.939 | -0.3846 | Yes |
| 91 | CDCA3 |  |  | 4874 | -0.940 | -0.3760 | Yes |
| 92 | CENPM |  |  | 4881 | -0.947 | -0.3685 | Yes |
| 93 | RFC3 |  |  | 4887 | -0.954 | -0.3608 | Yes |
| 94 | GINS2 |  |  | 4897 | -0.962 | -0.3537 | Yes |
| 95 | CDCA4 |  |  | 4902 | -0.967 | -0.3457 | Yes |
| 96 | AURKA |  |  | 4906 | -0.973 | -0.3374 | Yes |
| 97 | FBXO5 |  |  | 4910 | -0.978 | -0.3290 | Yes |
| 98 | DNAJC9 |  |  | 4916 | -0.982 | -0.3210 | Yes |
| 99 | H2AFZ |  |  | 4919 | -0.983 | -0.3124 | Yes |
| 100 | SNRPB |  |  | 4931 | -0.996 | -0.3054 | Yes |
| 101 | PSMC3IP |  |  | 4933 | -0.997 | -0.2965 | Yes |
| 102 | ATAD5 |  |  | 4934 | -0.998 | -0.2874 | Yes |
| 103 | BRCA1 |  |  | 4938 | -1.002 | -0.2788 | Yes |
| 104 | CTNNAL1 |  |  | 4945 | -1.007 | -0.2708 | Yes |
| 105 | MCM4 |  |  | 4947 | -1.011 | -0.2617 | Yes |
| 106 | LIMS1 |  |  | 4961 | -1.031 | -0.2548 | Yes |
| 107 | ATAD2 |  |  | 4980 | -1.058 | -0.2487 | Yes |
| 108 | UBE2T |  |  | 5000 | -1.091 | -0.2424 | Yes |
| 109 | MAD2L1 |  |  | 5007 | -1.105 | -0.2334 | Yes |
| 110 | FEN1 |  |  | 5014 | -1.113 | -0.2244 | Yes |
| 111 | TK1 |  |  | 5020 | -1.126 | -0.2151 | Yes |
| 112 | FAM111B |  |  | 5054 | -1.199 | -0.2106 | Yes |
| 113 | PCNA |  |  | 5060 | -1.214 | -0.2004 | Yes |
| 114 | MCM10 |  |  | 5066 | -1.222 | -0.1902 | Yes |
| 115 | ESCO2 |  |  | 5067 | -1.222 | -0.1791 | Yes |
| 116 | BRCA2 |  |  | 5069 | -1.222 | -0.1681 | Yes |
| 117 | CENPK |  |  | 5091 | -1.267 | -0.1606 | Yes |
| 118 | SLC20A1 |  |  | 5094 | -1.277 | -0.1493 | Yes |
| 119 | CDC6 |  |  | 5107 | -1.314 | -0.1396 | Yes |
| 120 | CCDC34 |  |  | 5111 | -1.316 | -0.1282 | Yes |
| 121 | ZWINT |  |  | 5115 | -1.321 | -0.1167 | Yes |
| 122 | PHLDA2 |  |  | 5155 | -1.458 | -0.1109 | Yes |
| 123 | TYMS |  |  | 5156 | -1.477 | -0.0974 | Yes |
| 124 | CENPU |  |  | 5159 | -1.486 | -0.0843 | Yes |
| 125 | CDK1 |  |  | 5164 | -1.512 | -0.0712 | Yes |
| 126 | GJB2 |  |  | 5199 | -1.658 | -0.0627 | Yes |
| 127 | CLSPN |  |  | 5202 | -1.663 | -0.0478 | Yes |
| 128 | RRM2 |  |  | 5232 | -1.875 | -0.0363 | Yes |
| 129 | TPM1 |  |  | 5254 | -2.143 | -0.0208 | Yes |
| 130 | TAGLN |  |  | 5285 | -3.021 | 0.0010 | Yes |
Table: GSEA details [plain text format]

  

Fig 2: FELDMAN\_2020\_S: Random ES distribution      
 Gene set null distribution of ES for **FELDMAN\_2020\_S**
